# Predictive powers of the Modified Early Warning Score and the National Early Warning Score in general ward patients who activated the medical emergency team

**DOI:** 10.1371/journal.pone.0233078

**Published:** 2020-05-14

**Authors:** Jee Hwan Ahn, Youn Kyung Jung, Ju-Ry Lee, You Na Oh, Dong Kyu Oh, Jin Won Huh, Chae-Man Lim, Younsuck Koh, Sang-Bum Hong

**Affiliations:** 1 Department of Pulmonary and Critical Care Medicine, Asan Medical Center, University of Ulsan College of Medicine, Seoul, Republic of Korea; 2 Medical Emergency Team, Asan Medical Center, Seoul, Republic of Korea; University of Pittsburgh, UNITED STATES

## Abstract

**Background:**

The current early warning scores may be insufficient for medical emergency teams (METs) to use in assessing the severity and the prognosis of activated patients. We evaluated the predictive powers of the Modified Early Warning Score (MEWS) and the National Early Warning Score (NEWS) for 28-day mortality and to analyze predictors of 28-day mortality in general ward patients who activate the MET.

**Methods:**

Adult general ward inpatients who activated the MET in a tertiary referral teaching hospital between March 2009 and December 2016 were included. The demographic and clinical characteristics and physiologic parameters at the time of MET activation were collected, and MEWS and NEWS were calculated.

**Results:**

A total of 6,729 MET activation events were analyzed. Patients who died within 28 days were younger (mean age 60 vs 62 years), were more likely to have malignancy (72% vs 53%), were more likely to be admitted to the medical department rather than the surgical department (93% vs 80%), had longer intervals from admission to MET activation (median, 7 vs 5 days), and were less likely to activate the MET during nighttime hours (5 PM to 8 AM) (61% vs 66%) compared with those who did not die within 28 days (*P* < 0.001 for all comparisons). The areas under the receiver operating characteristic curves of MEWS and NEWS for 28-day mortality were 0.58 (95% CI, 0.56–0.59) and 0.60 (95% CI, 0.59–0.62), which were inferior to that of the logistics regression model (0.73; 95% CI, 0.72–0.74; *P* < 0.001 for both comparisons).

**Conclusions:**

Both the MEWS and NEWS had poor predictive powers for 28-day mortality in patients who activated the MET. A new scoring system is needed to stratify the severity and prognosis of patients who activated the MET.

## Introduction

Patients in general wards can undergo acute deterioration, resulting in poor outcomes including unexpected intensive care unit (ICU) transfer, cardiac arrest, or death. Clinical deterioration is observed in 5%–9% of hospitalized patients in the general wards [1, 2]. Timely and proper management of acute deterioration events is important for patient safety. To manage unexpected deterioration in hospitalized patients, many hospitals have operated rapid response systems (RRSs) [3]. The RRS has two major axes—the afferent arm and the efferent arm [4]. The afferent arm identifies patients at risk of clinical deterioration and activates the efferent arm if necessary. The efferent arm, a medical emergency team (MET), examines the patients and intervenes in the treatment. Effective functioning of both arms is essential for the success of the RRS.

The early warning score (EWS) is a scoring system that assesses the risk of deterioration in hospitalized patients [5]. The EWS was developed based on the findings that abnormal changes in physiologic parameters such as vital signs or mental status often precede overt clinical deterioration by several hours [6–8]. Following the development of the RRS, a variety of EWS systems including the Modified Early Warning Score (MEWS) and the National Early Warning Score (NEWS) have been utilized in the afferent arm [8, 9]. However, in clinical practice, not all patients identified as high-risk through EWS systems receive higher levels of intervention by METs [10]. In some cases, low-risk patients are misidentified as high-risk due to limited discriminatory power of the EWS (false alarm), or high-risk patients do not receive adequate intervention due to a shortage of available medical resources.

The current EWS systems may be insufficient for METs to use in assessing the severity and the prognosis of activated patients [11]. Many screening tools have been developed to detect at-risk inpatients [12], but confirmatory tools with high specificity for the necessity of intervention and higher levels of care are not well-studied. We thus evaluated the predictive powers of MEWS and NEWS for 28-day mortality and identified the predictors for 28-day mortality in patients who activated the MET.

## Materials and methods

### Study patients

This retrospective cohort study was conducted at Asan Medical Center, a tertiary referral teaching hospital with approximately 100,000 adult inpatients per year. Among all MET activation events in adult (aged 18 years or more) general ward patients that occurred between March 2009 and December 2016, the first activation event was included in the study. Exclusion criteria were as follows: activation by cardiac arrest; activation only to aid in equipment manipulation or procedures; activation that resulted in a do-not-resuscitate order; activation with missing data needed for calculation of MEWS and NEWS. The protocols of this study were approved by the institutional review board of Asan Medical Center (IRB No. 2016–0857), which waived the need for informed consent considering the retrospective nature of the study. Data of all study patients were de-identified before analyses to secure patients' personal information.

### MET

We started operating an MET at our hospital for adult patients on a part-time basis (from 7 AM to 7 PM daily) in March 2008, and expanded its coverage to full-time in March 2009. The MET consists of 3 ICU staffs (intensivist), 4 ICU fellows, 2 internal medicine residents, and 9 dedicated nurses with experience in critical care. At least 1 intensivist or fellow, 1 resident, and 2 dedicated nurses work on every duty.

The MET was activated when 1) the measurements of a patient exceeded the pre-defined thresholds in the electronic medical record-based automatic screening system, 2) doctors or nurses called the MET for aid, or 3) code blue was announced for cardiopulmonary arrest, as published previously [13]. The electronic medical record-based screening system used single parameter triggering criteria, which were modified three times during the study period to improve its accuracy (**S1 Table**).

### Data collection

At the end of each MET activation, the MET nurse who participated in the activation event recorded the patient's data based on the medical record. Included data were as follows: age, sex, admission date, diagnosis at admission, comorbidities, date and time of MET activation, interval between admission and MET activation, clinical department at the time of activation, activation method (screening/calling), cause of activation, blood pressure, heart rate, respiratory rate, body temperature, level of consciousness (alert/confused/drowsy/unresponsive [ACDU] scale), oxygen supply, peripheral oxygen saturation at the time of activation, do-not-resuscitate order before and after MET activation, MET activities, and the result of MET activation (stay in general ward/transfer to ICU). The causes of activation were categorized into shock, respiratory distress, altered mental status, metabolic acidosis, or others (including hypotension and arrhythmias). In addition, ICU discharge date, hospital discharge date, and date of death in the patients with MET activation were collected annually. Variables that were not documented at the time of MET activation were treated as missing values. The primary outcome was mortality within 28 days after MET activation.

### Statistical analysis

Data are presented as either mean with standard deviation or median with interquartile range (IQR). For continuous variables, the range of each quartile and the number of patients in each quartile are presented together. MEWS and NEWS were calculated from data at the time of MET activation. For the level of consciousness collected in the ACDU scale, confused and drowsy mental status were treated as 1 point of MEWS and unresponsive was treated as 3 points of MEWS based on the previous study [14]. Mental status other than alert was treated as 3 points of NEWS [15]. The variables were compared between patients who survived for 28 days after MET activation and those who did not using the Student's t-test or the Mann-Whitney *U* test for continuous variables and the chi-square test for categorical variables. If the patient survived to discharge within 28 days after MET activation, the patient was considered to survive for 28 days. To report predictive performance of MEWS and NEWS at the critical threshold, the cutoff values of MEWS ≥ 5 and NEWS ≥ 7 were used [8, 9]. The multivariable logistic regression model was derived from the collected variables using stepwise backward selection. Continuous variables were converted to categorical variables according to their quartiles and entered into the model. The goodness-of-fit of the final regression model was assessed using the chi-square and the Hosmer–Lemeshow tests. The areas under the receiver operating characteristic curve (AUROC) for the primary outcome were compared among MEWS, NEWS, and the logistic regression model by the DeLong's test [16]. Two-sided *P* values of less than 0.05 were considered statistically significant. All analyses were performed using IBM SPSS Statistics version 21 (IBM Corp., Armonk, NY, USA).

## Results

A total of 11,102 MET activation events had occurred during the study period; the median annual number of activations was 17.3 (interquartile range [IQR], 16.1–19.4) per 1,000 admissions. After excluding activation by cardiac arrest (n = 745), activation to assist with equipment manipulation or procedures (n = 508), activation ended in do-not-resuscitate order (n = 2,266), and activation with insufficient data for calculating MEWS and NEWS (n = 854), the remaining 6,729 MET activation events were included in the analysis (**S1 Fig**).

As a result of MET activation, 2,274 patients (34%) were transferred to the ICU; 1,717 patients (26%) died within 28 days of the activation, and in-hospital mortalities occurred in 2,111 patients (31%). **Table 1** shows the demographic and activation characteristics of the patients according to 28-day mortality. The patients who died within 28 days of activation (non-survivors) were younger (mean age 60 vs 62 years), were more likely to have malignancy (72% vs 53%), were more likely to be admitted to the medical department rather than the surgical department (93% vs 80%), had longer intervals from admission to MET activation (median, 7 vs 5 days), and were less likely to activate the MET during nighttime hours (5 PM to 8 AM) (61% vs 66%) compared with those who did not die within 28 days (survivors) (*P* < 0.001 for all comparisons). The physiologic parameters of the patients at the time of MET activation are presented in **Table 2**. The non-survivors had higher heart rate (114 vs 107 beats per minute) and respiratory rate (27 vs 25 breaths per minute), were less likely to be alert (63% vs 70%), and more likely to be given oxygen therapy (79% vs 66%) than the survivors (*P* < 0.001 for all comparisons). The treatment outcomes are shown in **Table 3**.

**Table 1:** Demographic and clinical characteristics of patients who activated medical emergency team depending on 28-day mortality.

Characteristics	Survivor, n = 5,012	Non-survivor, n = 1,717	*P*
Age, years, mean ± SD	62.0 ± 15.0	59.9 ± 14.1	< 0.001
< 54, n (%)	1,285 (26)	527 (31)	
54–63, n (%)	1,126 (22)	447 (26)	
64–72, n (%)	1,281 (26)	425 (25)	
≥ 73, n (%)	1,320 (26)	318 (18)	
Male sex, n (%)	3,038 (61)	1,111 (65)	0.003
Comorbidities, n (%)			
No comorbidities	466 (9)	61 (4)	< 0.001
Solid malignancy	1,979 (40)	876 (51)	< 0.001
Hematologic malignancy	699 (14)	381 (22)	< 0.001
Chronic lung diseases	654 (13)	214 (13)	0.532
Cardiovascular diseases	2,181 (44)	657 (38)	< 0.001
Hepatobiliary diseases	596 (12)	346 (20)	< 0.001
Stroke	648 (13)	115 (7)	< 0.001
Chronic kidney disease	331 (7)	86 (5)	0.018
Diabetes mellitus	1,279 (26)	364 (21)	< 0.001
History of transplantation	148 (3)	66 (4)	0.069
Medical department, n (%)	4,020 (80)	1,589 (93)	< 0.001
Time to activation from admission, days, median (IQR)	5 (1–14)	7 (2–18)	< 0.001
< 2, n (%)	1,350 (27)	334 (20)	
2–5, n (%)	1,352 (27)	417 (24)	
6–15, n (%)	1,206 (24)	472 (28)	
≥ 16, n (%)	1,104 (22)	494 (29)	
Activation during nighttime hours, n (%)	3,325 (66)	1,053 (61)	< 0.001
Activation types, n (%)			< 0.001
Screening	2,543 (51)	963 (56)	
Calling	2,469 (49)	754 (44)	
Activation causes, n (%)			< 0.001
Shock	1,008 (20)	334 (20)	
Respiratory distress	2,516 (50)	964 (56)	
Altered mental status	296 (6)	116 (7)	
Metabolic acidosis	259 (5)	142 (8)	
Others	933 (19)	161 (9)	

Abbreviations: SD, standard deviation; IQR, interquartile range

**Table 2:** Physiologic parameters and early warning scores at the time of medical emergency team activation according to 28-day mortality.

Variables	Survivor, n = 5,012	Non-survivor, n = 1,717	*P*
Systolic blood pressure, mm Hg, mean ± SD	114.5 ± 31.7	114.0 ± 28.6	0.545
< 91, n (%)	1,352 (27)	397 (23)	
91–112, n (%)	1,190 (24)	451 (26)	
113–135, n (%)	1,202 (24)	456 (27)	
≥ 136, n (%)	1,268 (25)	413 (24)	
Diastolic blood pressure, mm Hg, mean ± SD	69.2 ± 19.3	69.7 ± 18.6	0.347
< 56, n (%)	1,304 (26)	395 (23)	
56–69, n (%)	1,289 (26)	439 (26)	
70–81, n (%)	1,199 (24)	438 (26)	
≥ 82, n (%)	1,220 (24)	445 (26)	
Heart rate, beats/min, mean ± SD	106.6 ± 26.3	114.3 ± 25.5	< 0.001
< 91, n (%)	1,419 (28)	299 (17)	
91–108, n (%)	1,274 (25)	395 (23)	
109–125, n (%)	1,227 (25)	483 (28)	
≥ 126, n (%)	1,092 (22)	540 (32)	
Respiratory rate, breaths/min, mean ± SD	25.0 ± 7.4	26.5 ± 7.1	< 0.001
< 21, n (%)	1,776 (35)	396 (23)	
21–24, n (%)	1,123 (22)	393 (23)	
25–30, n (%)	1,145 (23)	512 (30)	
≥ 31, n (%)	968 (19)	416 (24)	
Body temperature, °C, mean ± SD	37.15 ± 0.93	37.05 ± 0.91	< 0.001
< 36.5, n (%)	1,221 (24)	480 (28)	
36.5–36.9, n (%)	1,395 (28)	498 (29)	
37.0–37.7, n (%)	1,178 (24)	369 (21)	
≥ 37.8, n (%)	1,218 (24)	370 (22)	
Mental status, n (%)			< 0.001
Alert	3,493 (70)	1,080 (63)	
Confused	203 (4)	106 (6)	
Drowsy	652 (13)	248 (14)	
Unresponsive	664 (13)	283 (17)	
SpO_2_, %, mean ± SD	93.0 ± 8.9	92.8 ± 7.9	< 0.001
< 92, n (%)	1,346 (27)	505 (29)	
92–95, n (%)	1,200 (24)	461 (27)	
96–98, n (%)	1,536 (31)	469 (27)	
99–100, n (%)	930 (19)	282 (16)	
Oxygen supply, n (%)	3,291 (66)	1,348 (79)	< 0.001
Modified Early Warning Score, mean ± SD	4.5 ± 2.1	5.1 ± 2.2	< 0.001
National Early Warning Score, mean ± SD	7.8 ± 3.2	9.0 ± 3.1	< 0.001

Abbreviations: SD, standard deviation

**Table 3:** Treatment outcomes of the study patients depending on 28-day mortality.

Outcomes	Survivor, n = 5,012	Non-survivor, n = 1,717	*P*
ICU transfer, n (%)	1,652 (33)	622 (36)	0.014
ICU length of stay, days, median (IQR)	3 (1–8)	3 (1–8)	0.443
Hospital length of stay, days, median (IQR)	27 (14–53)	17 (8–30)	< 0.001
Post-activation hospital length of stay, days, median (IQR)	18 (9–38)	6 (2–13)	< 0.001

Abbreviations: ICU, intensive care unit; IQR, interquartile range

**Fig 1:**
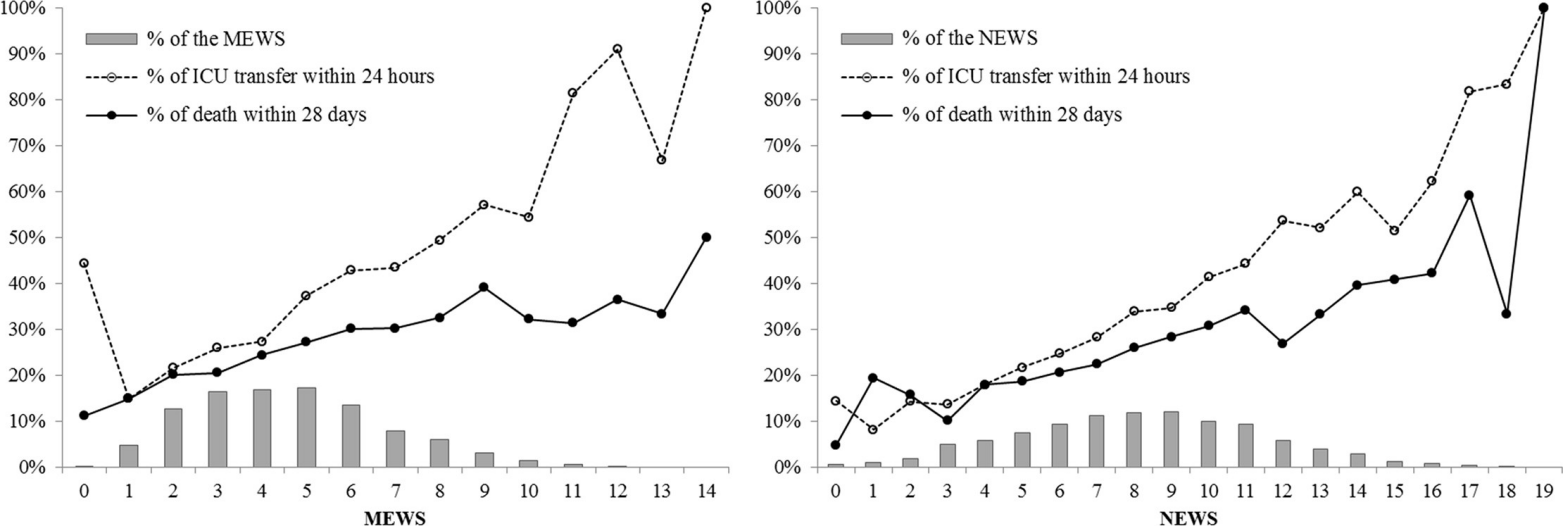
Distribution of early warning scores and outcomes based on the scores in patients who activated the medical emergency team. Dashed lines showed the rates of ICU transfer within 24 hours after the activation and solid lines showed 28-day mortality at each score. MEWS, Modified Early Warning Score; NEWS, National Early Warning Score; ICU, intensive care unit.

The non-survivors had significantly higher scores in both MEWS and NEWS (5.1 ± 2.2 and 9.0 ± 3.1, respectively) than did the survivors (4.5 ± 2.1 and 7.8 ± 3.2, respectively) (*P* < 0.001 in both comparisons). The distribution of MEWS and NEWS at the time of MET activation and ICU transfer as well as 28-day mortality rates at each score are plotted in **Fig 1**. When using a cutoff value of ≥ 5, MEWS had a sensitivity of 58.2% (95% CI, 55.8%–60.5%) and a specificity of 53.7% (95% CI, 52.3%–55.0%) for 28-day mortality (**Table 4**). Of the 3,407 patients with MEWS < 5, 827 patients (24%) were transferred to the ICU and 718 patients (21%) died within 28 days. When using a cutoff value of ≥ 7, NEWS had a sensitivity of 79.2% (95% CI, 77.2%–81.1%) and a specificity of 34.2% (95% CI, 32.9%–35.5%) for 28-day mortality. Among the 2,071 patients with NEWS < 7, 404 patients (20%) were transferred to the ICU and 357 patients (17%) died within 28 days. The AUROC of MEWS and NEWS for 28-day mortality were 0.58 (95% CI, 0.56–0.59) and 0.60 (95% CI, 0.59–0.62), respectively. The multivariable logistic regression model for 28-day mortality was derived from the clinical and physiologic variables at MET activation (**Table 5**; *P* < 0.001 by chi-square test, *P* = 0.143 by Hosmer–Lemeshow test). Age, comorbidities, medical department, days from admission, activation during daytime hours, activation by screening, activation causes, vital signs, mental status, and oxygen supply were identified as the predictors of 28-day mortality. The AUROC of the logistic regression model was 0.73 (95% CI based on bootstrap resampling, 0.72–0.74), which was superior to those of MEWS and NEWS (*P* < 0.001 for both comparisons, **S2 Fig**).

**Table 4:** Predictive accuracy of the early warning scores for 28-day mortality in patients who activated the medical emergency team.

Statistics	MEWS ≥ 5, n = 3,322	NEWS ≥ 7, n = 4,658
Sensitivity (95% CI)	58.2% (55.8%–60.5%)	79.2% (77.2%–81.1%)
Specificity (95% CI)	53.7% (52.3%–55.0%)	34.2% (32.9%–35.5%)
Positive predictive value (95% CI)	30.1% (29.0%–31.1%)	29.2% (28.6%–29.9%)
Negative predictive value (95% CI)	78.9% (77.9%–79.9%)	82.8% (81.3%–84.1%)
Positive likelihood ratio (95% CI)	1.26 (1.19–1.32)	1.20 (1.17–1.24)
Negative likelihood ratio (95% CI)	0.78 (0.73–0.83)	0.61 (0.55–0.67)
Accuracy (95% CI)	54.8% (53.6%–56.0%)	45.7% (44.5%–46.9%)

Abbreviations: MEWS, Modified Early Warning Score; NEWS, National Early Warning Score; CI, confidence interval

**Table 5:** Multivariable logistic regression model for 28-day mortality after medical emergency team activation.

Variables	Adjusted OR	95% CI	*P*
Age, years			0.042
< 54	1.00		
54–63	0.97	0.82–1.14	0.707
64–72	0.90	0.76–1.06	0.214
≥ 73	0.78	0.65–0.93	0.007
Comorbidities			
No comorbidities	0.67	0.49–0.91	0.010
Solid malignancy	1.95	1.69–2.25	< 0.001
Hematologic malignancy	1.73	1.43–2.08	< 0.001
Hepatobiliary diseases	1.85	1.57–2.18	< 0.001
Stroke	0.57	0.45–0.72	< 0.001
Diabetes mellitus	0.85	0.74–0.99	0.034
Medical department	2.92	2.37–3.60	< 0.001
Time to activation from admission, days			< 0.001
< 2	1.00		
2–5	1.21	1.02–1.44	0.034
6–15	1.50	1.27–1.79	< 0.001
≥ 16	1.71	1.43–2.04	< 0.001
Activation during nighttime hours	0.84	0.74–0.95	0.005
Activation by calling	0.88	0.78–1.00	0.049
Activation causes			< 0.001
Shock	1.00		
Respiratory distress	1.07	0.87–1.30	0.540
Altered mental status	1.14	0.84–1.56	0.406
Metabolic acidosis	1.66	1.25–2.20	0.001
Others	0.66	0.52–0.82	< 0.001
Systolic blood pressure, mm Hg			0.029
< 91	1.00		
91–112	0.93	0.77–1.12	0.442
113–135	0.90	0.74–1.11	0.329
≥ 136	0.75	0.61–0.93	0.008
Heart rate, beats/min			< 0.001
< 91	1.00		
91–108	1.29	1.08–1.55	0.006
109–125	1.53	1.28–1.83	< 0.001
≥ 126	1.88	1.55–2.27	< 0.001
Respiratory rate, breaths/min			< 0.001
< 21	1.00		
21–24	1.36	1.15–1.63	0.001
25–30	1.67	1.40–1.99	< 0.001
≥ 31	1.57	1.29–1.90	< 0.001
Body temperature, °C			< 0.001
< 36.5	1.00		
36.5–36.9	0.94	0.80–1.10	0.403
37.0–37.7	0.77	0.64–0.91	0.002
≥ 37.8	0.63	0.52–0.75	< 0.001
Mental status			< 0.001
Alert	1.00		
Confused	1.71	1.31–2.23	< 0.001
Drowsy	1.30	1.09–1.55	0.004
Unresponsive	1.69	1.40–2.04	< 0.001
Oxygen supply	1.77	1.52–2.07	< 0.001

Abbreviations: OR, odds ratio; CI, confidence interval

## Discussion

We investigated the predictive powers of MEWS and NEWS for 28-day mortality and identified the predictors for 28-day mortality in general ward patients who activated the MET. The predictive powers based on the AUROC were poor in both MEWS and NEWS. Approximately 20% of patients who had EWS lower than critical thresholds (MEWS < 5 or NEWS < 7) needed to be transferred to the ICU or died within 28 days of MET activation. Our results suggest that it may not be appropriate for the MET to assess the severity of patients and determine the level of treatment solely based on MEWS or NEWS.

Shappell et al. and Fernando et al. have evaluated the predictive power of EWS for the treatment outcome in patients with MET activation. Shappell et al. developed logistic regression and machine learning models for predicting in-hospital mortality and compared their discriminatory powers to that of NEWS using the national MET registry from 274 hospitals in the United States [11]. The authors reported that the discriminatory power of NEWS was poor (AUROC, 0.66) and was significantly lower than that of the logistic regression model (AUROC, 0.73). Our results were similar in that the predictive power of the logistic regression model was significantly higher than that of NEWS. Fernando et al. compared the predictive accuracy for in-hospital mortality between the Hamilton Early Warning Score (HEWS) and the National Early Warning Score 2 (NEWS2) at MET activation [17], and showed that HEWS and NEWS2 both had comparably high accuracy (AUROC, 0.76 and 0.72, respectively). This result is different from the results of our current study, but the AUROC values in the two studies cannot be directly compared because the primary outcome (28-day mortality vs in-hospital mortality) and the EWS used (NEWS vs NEWS2) were different. In addition, difference in the severity of the patients may have resulted in such difference—whereas 62.2% of patients in Fernando et al. had NEWS2 of 5 or more, 85.7% of our patients had NEWS of 5 or more.

We identified multiple clinical characteristics as well as physiologic parameters at the time of MET activation that were significantly associated with 28-day mortality. Among them, the findings on vital signs, mental status, and oxygen supply are consistent with those of previous studies regarding EWS or MET [9, 11, 18, 19]. Our result is in line with the results of Shappell et al. in that patients in the medical department or those with longer hospitalization duration had higher odds of 28-day mortality [11]. Some results are not in line with those from previous studies. In our patients, peripheral oxygen saturation was not associated with 28-day mortality, which may be because it can be rapidly corrected with oxygen therapy by general ward physicians. Several studies reported that older age at MET activation, especially 75 years or more, was associated with higher mortality [11, 20, 21]. In contrast, in the current study, patients aged 73 years or more had lower odds of 28-day mortality than those aged 53 years or less. Also, MET activation during nighttime was associated with lower 28-day mortality compared with daytime activation, which is in conflict with previous studies [21, 22]. This may be attributable to the difference in the activation method of the MET: while the MET was activated only by calling from the ward staff in those studies, MET activation in our study was based on electronic medical record-based screening as well as calling. Our results suggest that electronic medical record-based screening and subsequent intervention by the MET could compensate for relative insufficiency in the number and experience of general ward medical staffs during nighttime.

The current study is distinct from previous studies investigating the prognostic accuracy of the EWS in that patients with do-not-resuscitate orders were excluded. An important aspect of MET activities is aiding in the decision on a do-not-resuscitate order and providing end-of-life care. However, the decision on a do-not-resuscitate order is mostly based on medical futility and patient autonomy [23], not on the severity of the patient. Thus, if patients with do-not-resuscitate orders are included when evaluating the predictive power of EWS, the results may be inadequate for application in clinical settings in which an MET should determine the level of intervention based on the severity of the patient. We therefore tried to evaluate the actual performance of MEWS and NEWS by excluding patients with do-not-resuscitate orders.

This study is limited in that EWS was only calculated with the measurements at the time of MET activation. The predictive power of EWS may have been higher if we had used EWS at the beginning of acute deterioration before activation of the MET or the worst EWS during the episode of acute deterioration. Nevertheless, as the goal of this study was to investigate whether EWS at the time of activation was useful for the MET in assessing the severity and prognosis of patients, it was appropriate to use EWS at the time of MET activation. Also, we used the ACDU scale and not the alert/voice/pain/unresponsive (AVPU) scale in categorizing the level of consciousness. At our center, the ACDU scale has been used to detect deterioration in mental status earlier. There may be some difference between the EWS calculated based on the ACDU scale and the actual EWS. However, we converted the ACDU scale to the AVPU scale in the EWS based on previously reported data [14], and the higher adjusted odds ratio of the confused patients to the alert patients in our data also supports the validity of this conversion. Thirdly, the study results may have limited generalizability because this study was conducted in a single center. Specifically, more than half of our patients (57.8%) had malignancies, which is a higher proportion than those of MET cohorts in previous studies [11, 17]. To assess the outcome of the MET activation event itself rather than the outcome of underlying malignancy, we defined the primary outcome as 28-day mortality from MET activation. Lastly, the original goal of the EWS is not to stratify the risk of death in patients with MET activation, but to herald acute deterioration and to trigger clinical responses accordingly in general in-patients. However, if a MET can accurately stratify the severity or prognosis of the patient at activation, it will help to decide the level of intervention, treatment priority, and allocation of medical resources. Because it is effective if a MET can utilize the already calculated EWS to determine the severity or prognosis of the patient, we investigated the prognostic power of the MEWS and NEWS at MET activation.

## Conclusions

Both the MEWS and NEWS had poor predictive powers for 28-day mortality in patients who activated the MET, suggesting that MEWS and NEWS may be insufficient for stratifying the severity and prognosis of general ward patients who activate the MET. This warrants the need for the development of a new, practical scoring system for use by the MET in deciding the optimal treatment of patients and the allocation of medical resources.

## Supporting information

S1 Table Screening criteria based on electronic medical record for triggering medical emergency team activation. (DOCX)

S1 Fig Flowchart of the inclusion and exclusion of the medical emergency team activation events. MET, medical emergency team.
(TIF)

S2 Fig Receiver operating characteristic curves of discrimination for 28-day mortality. ROC, receiver operating characteristic; MEWS, Modified Early Warning Score; NEWS, National Early Warning Score.
(TIF)

## References

[1] Buist M, Bernard S, Nguyen TV, Moore G, Anderson J. Association between clinically abnormal observations and subsequent in-hospital mortality: a prospective study. Resuscitation. 2004; 62(2):137–41. 10.1016/j.resuscitation.2004.03.005 15294398

[2] Bell MB, Konrad D, Granath F, Ekbom A, Martling CR. Prevalence and sensitivity of MET-criteria in a Scandinavian University Hospital. Resuscitation. 2006; 70(1):66–73. 10.1016/j.resuscitation.2005.11.011 16757089

[3] Jones DA, DeVita MA, Bellomo R. Rapid-response teams. N Engl J Med. 2011; 365(2):139–46. 10.1056/NEJMra0910926 21751906

[4] Devita MA, Bellomo R, Hillman K, Kellum J, Rotondi A, Teres D, et al Findings of the first consensus conference on medical emergency teams. Crit Care Med. 2006; 34(9):2463–78. 10.1097/01.CCM.0000235743.38172.6E 16878033

[5] Morgan RJM, Williams F, Wright MM. An early warning scoring system for detecting developing critical illness. Clin Intensive Care. 1997; 8:100.

[6] Kause J, Smith G, Prytherch D, Parr M, Flabouris A, Hillman K. A comparison of antecedents to cardiac arrests, deaths and emergency intensive care admissions in Australia and New Zealand, and the United Kingdom—the ACADEMIA study. Resuscitation. 2004; 62(3):275–82. 10.1016/j.resuscitation.2004.05.016 15325446

[7] Churpek MM, Yuen TC, Huber MT, Park SY, Hall JB, Edelson DP. Predicting cardiac arrest on the wards: a nested case-control study. Chest. 2012; 141(5):1170–6. 10.1378/chest.11-1301 22052772 PMC3342781

[8] Subbe CP, Kruger M, Rutherford P, Gemmel L. Validation of a modified Early Warning Score in medical admissions. QJM. 2001; 94(10):521–6. 10.1093/qjmed/94.10.521 11588210

[9] Royal College of Physicians. National Early Warning Score (NEWS): standardising the assessment of acute-illness severity in the NHS Report of a working party. London: RCP; 2012.

[10] Subramaniam A, Botha J, Tiruvoipati R. The limitations in implementing and operating a rapid response system. Intern Med J. 2016; 46(10):1139–45. 10.1111/imj.13042 26913367

[11] Shappell C, Snyder A, Edelson DP, Churpek MM, American Heart Association's Get With The Guidelines-Resuscitation I. Predictors of In-Hospital Mortality After Rapid Response Team Calls in a 274 Hospital Nationwide Sample. Crit Care Med. 2018; 46(7):1041–8. 10.1097/CCM.0000000000002926 29293147 PMC6044728

[12] Smith GB, Prytherch DR, Meredith P, Schmidt PE, Featherstone PI. The ability of the National Early Warning Score (NEWS) to discriminate patients at risk of early cardiac arrest, unanticipated intensive care unit admission, and death. Resuscitation. 2013; 84(4):465–70. 10.1016/j.resuscitation.2012.12.016 23295778

[13] Huh JW, Lim CM, Koh Y, Lee J, Jung YK, Seo HS, et al Activation of a medical emergency team using an electronic medical recording-based screening system. Crit Care Med. 2014; 42(4):801–8. 10.1097/CCM.0000000000000031 24335439

[14] McNarry AF, Goldhill DR. Simple bedside assessment of level of consciousness: comparison of two simple assessment scales with the Glasgow Coma scale. Anaesthesia. 2004; 59(1):34–7. 10.1111/j.1365-2044.2004.03526.x 14687096

[15] Royal College of Physicians. National Early Warning Score (NEWS) 2: Standardising the assessment of acute-illness severity in the NHS Updated report of a working party. London: RCP; 2017.

[16] DeLong ER, DeLong DM, Clarke-Pearson DL. Comparing the areas under two or more correlated receiver operating characteristic curves: a nonparametric approach. Biometrics. 1988; 44(3):837–45. 3203132

[17] Fernando SM, Fox-Robichaud AE, Rochwerg B, Cardinal P, Seely AJE, Perry JJ, et al Prognostic accuracy of the Hamilton Early Warning Score (HEWS) and the National Early Warning Score 2 (NEWS2) among hospitalized patients assessed by a rapid response team. Crit Care. 2019; 23(1):60 10.1186/s13054-019-2355-3 30791952 PMC6385382

[18] Smith GB, Prytherch DR, Schmidt PE, Featherstone PI. Review and performance evaluation of aggregate weighted 'track and trigger' systems. Resuscitation. 2008; 77(2):170–9. 10.1016/j.resuscitation.2007.12.004 18249483

[19] Churpek MM, Yuen TC, Winslow C, Robicsek AA, Meltzer DO, Gibbons RD, et al Multicenter development and validation of a risk stratification tool for ward patients. Am J Respir Crit Care Med. 2014; 190(6):649–55. 10.1164/rccm.201406-1022OC 25089847 PMC4214112

[20] Tirkkonen J, Setala P, Hoppu S. Characteristics and outcome of rapid response team patients ≥75 years old: a prospective observational cohort study. Scand J Trauma Resusc Emerg Med. 2017; 25(1):77 10.1186/s13049-017-0423-8 28778172 PMC5544988

[21] Fernando SM, Reardon PM, McIsaac DI, Eagles D, Murphy K, Tanuseputro P, et al Outcomes of Older Hospitalized Patients Requiring Rapid Response Team Activation for Acute Deterioration. Crit Care Med. 2018; 46(12):1953–60. 10.1097/CCM.0000000000003442 30234523

[22] Fernando SM, Reardon PM, Bagshaw SM, Scales DC, Murphy K, Shen J, et al Impact of nighttime Rapid Response Team activation on outcomes of hospitalized patients with acute deterioration. Crit Care. 2018; 22(1):67 10.1186/s13054-018-2005-1 29534744 PMC5851273

[23] Jonsen AR, Siegler M, Winslade WJ. Clinical Ethics: A Practical Approach to Ethical Decisions in Clinical Medicine. 8th ed New York: McGraw-Hill Education; 2015.

