# Supplementary material for: Predictive powers of the Modified Early Warning Score and the National Early Warning Score in general ward patients who activated the medical emergency team
Source: PLoS One. 2020 May 14;15(5):e0233078. doi: 10.1371/journal.pone.0233078 (PMC7224474; doi:10.1371/journal.pone.0233078)
Supplement: S1 Table — (DOCX) [file pone.0233078.s001.docx]

| **S1 Table.** Screening criteria based on electronic medical record for triggering medical emergency team activation | | | | |
| --- | --- | --- | --- | --- |
|  |  |  |  |  |
| Systolic blood pressure < 86 mm Hg |  |  |  |  |
| Respiratory rate > 27 or < 8 breaths/minute | |  |  |  |
| Unexplained heart rate > 140 or < 40 beats/minute | | |  |  |
| Unexplained metabolic acidosis (pH < 7.3 or total CO_2_ < 12 mmol/L) or lactate > 2 mmol/L | | | | |
| PaCO_2_ > 50 mm Hg or PaO_2_ < 55 mm Hg |  |  |  |  |
| Applying O_2_ > 9 L/minute or FiO_2_ > 35% with Venturi mask | | |  |  |
| Sudden mental change or unexplained agitation | |  |  |  |
