# Supplementary figures and images for: Predictive powers of the Modified Early Warning Score and the National Early Warning Score in general ward patients who activated the medical emergency team
Source: PLoS One. 2020 May 14;15(5):e0233078. doi: 10.1371/journal.pone.0233078 (PMC7224474; doi:10.1371/journal.pone.0233078)

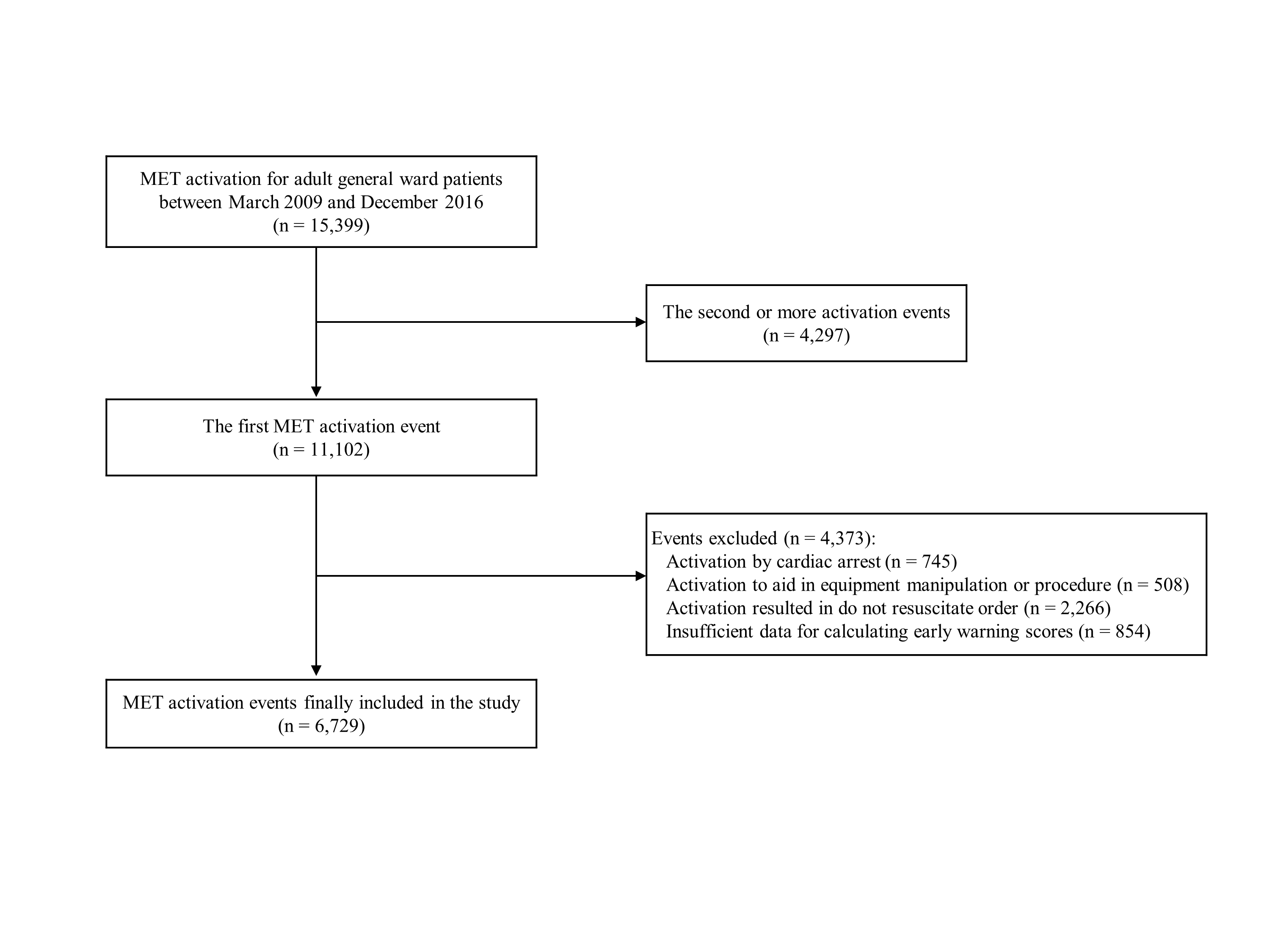

Supplement: S1 Fig — MET, medical emergency team. (TIF) [file pone.0233078.s002.TIF]

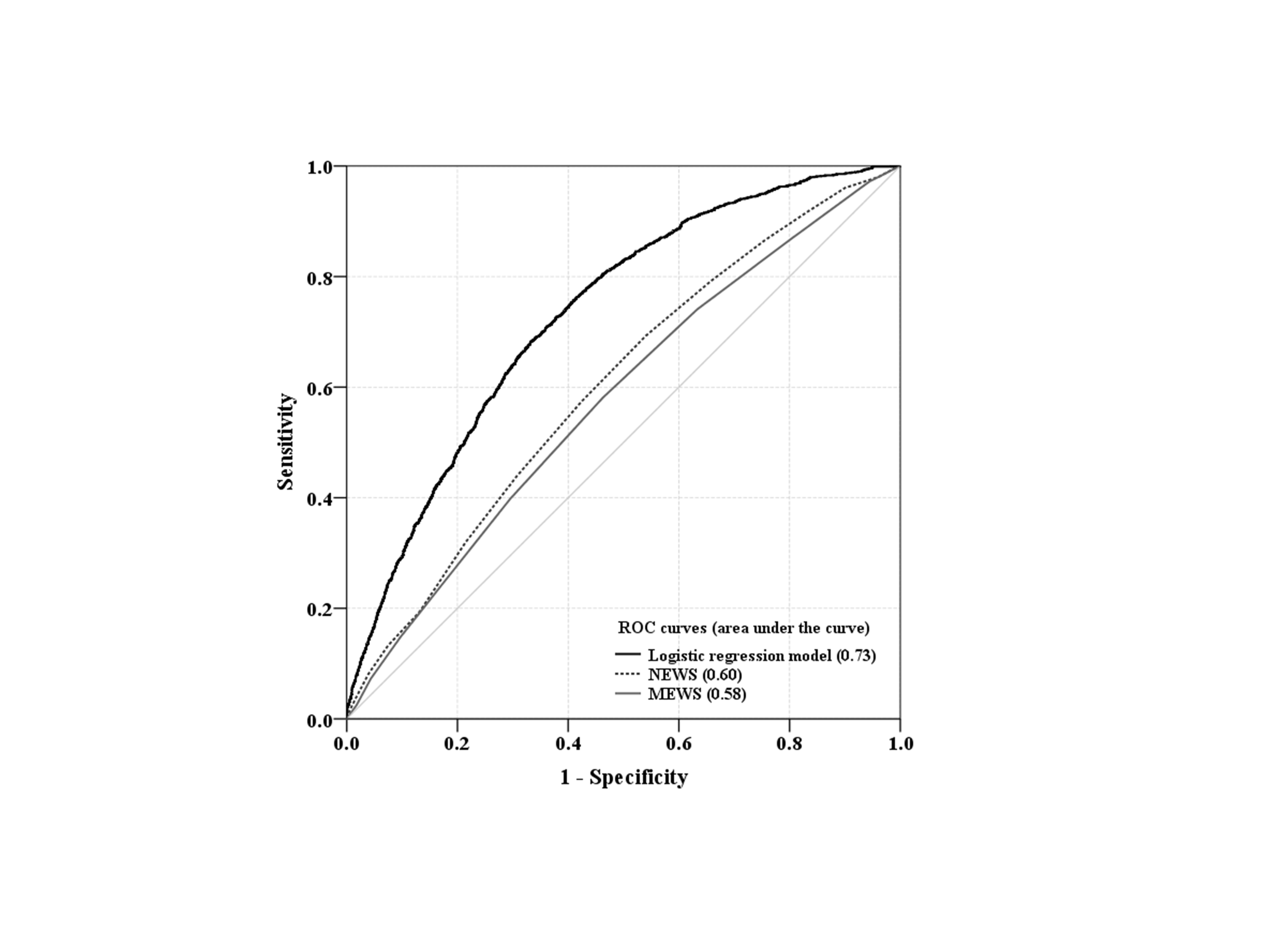

Supplement: S2 Fig — ROC, receiver operating characteristic; MEWS, Modified Early Warning Score; NEWS, National Early Warning Score. (TIF) [file pone.0233078.s003.TIF]
